# Supplementary material for: Quantitative comparative analysis of human erythrocyte surface proteins between individuals from two genetically distinct populations
Source: Commun Biol. 2019 Sep 20;2:350. doi: 10.1038/s42003-019-0596-y (PMC6754445; doi:10.1038/s42003-019-0596-y)
Supplement: Supplementary file 1 — Supplementary Information [file 42003_2019_596_MOESM1_ESM.pdf]

Supplementary figure 1

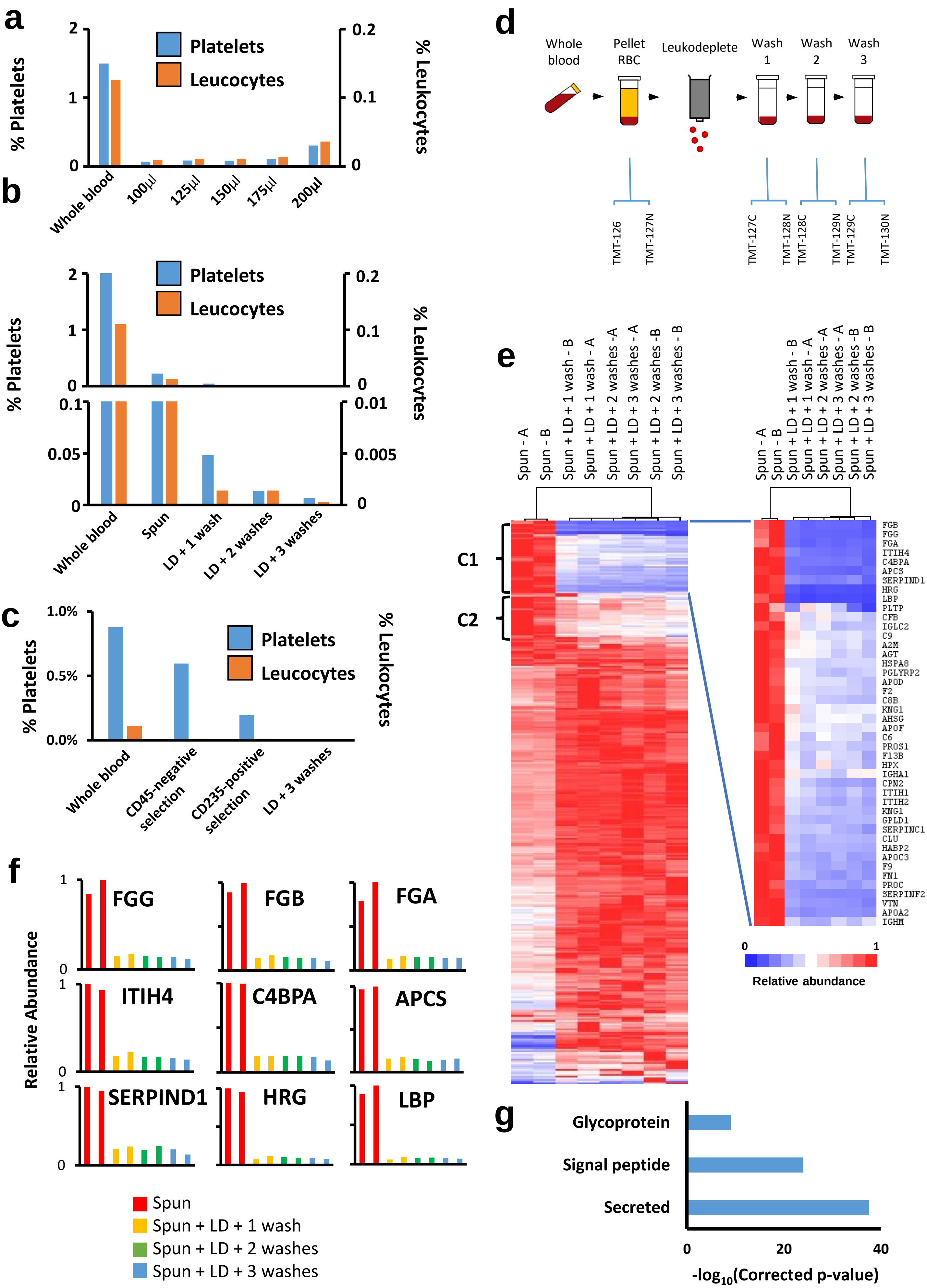

Supplementary Figure 1. An optimised RBC surface protein enrichment protocol. (a) Contamination of enriched RBC by platelets and leukocytes after microcentrifugation. 1 ml of whole blood was centrifuged at 1,000 *g* for 5 min in a 1.5 ml Eppendorf tube. A 200 µl pipette tip was used to withdraw the indicated volume of packed erythrocytes from the very bottom of the tube, and contamination was assessed by flow cytometry using CD61 as a platelet marker and CD45 as a leukocyte marker. Data are the average of two independent experiments. **(b)** RBC enriched from whole blood using the method indicated were similarly assessed for platelet and leukocyte contamination. 'Spun' indicates centrifugation as described in (a) followed by removal of 150 µl of packed erythrocytes. Leukodepletion (LD) used a Plasmodipur filter on spun populations of RBC as described in the Methods section, with 1-3 washes. The bottom panel shows an enlargement of the top graph, indicating that after leukodepletion and 3 washes, there were <0.001% leukocytes and <0.01% platelets. Data are the average of two independent experiments. **(c)** Comparison of leukocyte and platelet depletion methods using MACS microbeads with the optimised microcentrifugation / leukodepletion / wash protocol. **(d)** Schematic of the experimental workflow. **(e)** Hierarchical cluster analysis of proteins quantified in biological duplicate from the indicated RBC preparations described in (b), using signal:noise data normalised to a maximum of 1 for each protein from TMT-based proteomics. An enlargement of the subcluster C1 is shown in the right panel. **(f)** Example plots of the relative abundance of individual proteins in cluster C1. **(g)** Application of DAVID software identified enrichment of secreted glycoproteins amongst proteins in clusters C1 and C2, compared to all quantified proteins, indicative of serum contaminants.

Supplementary figure 2

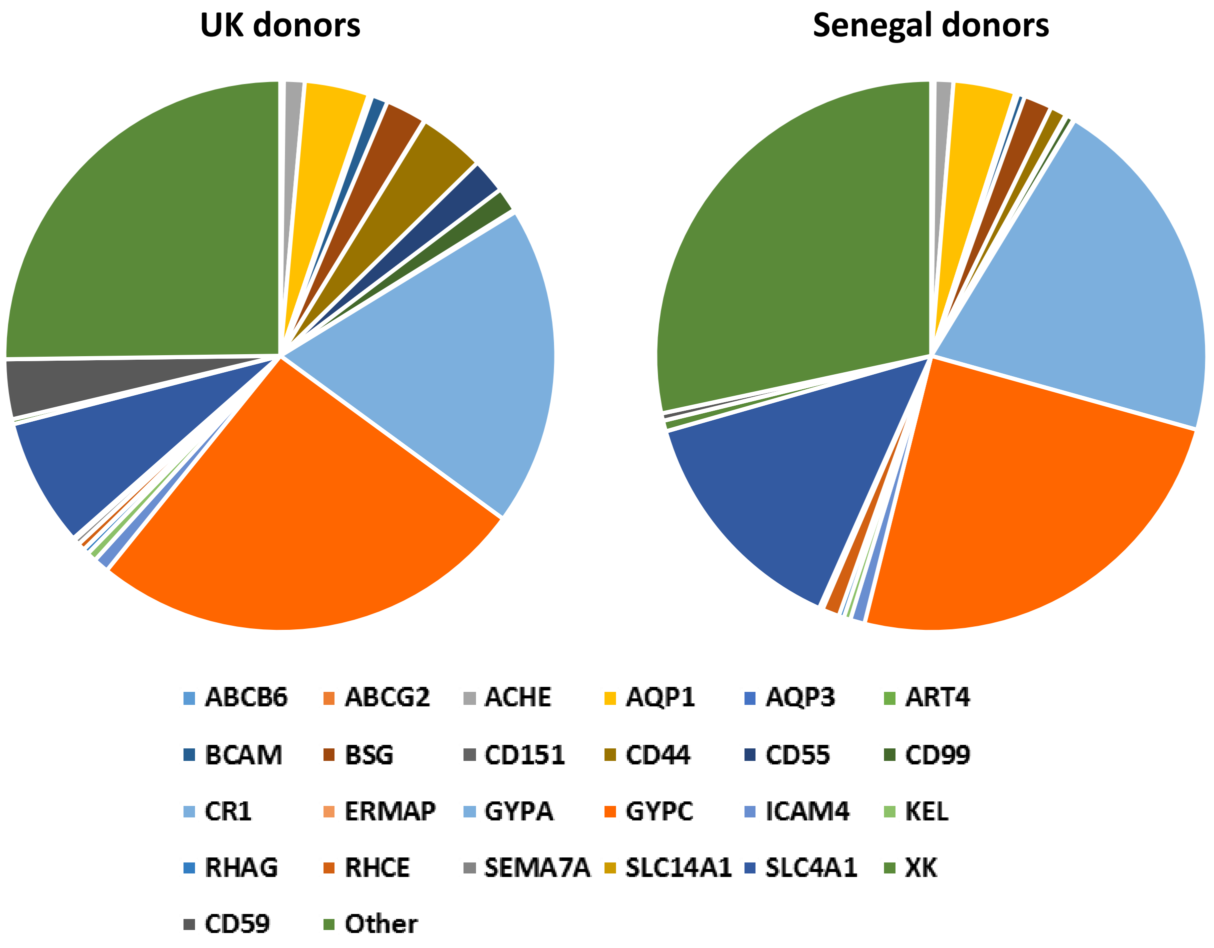

Supplementary Figure 2. Known blood group proteins constitute the majority of the RBC surface proteome. iBAQ abundances of all 25 quantified blood group proteins as a proportion of the total RBC surface proteome, in both populations. See also Supplementary Data 4.

Supplementary figure 3

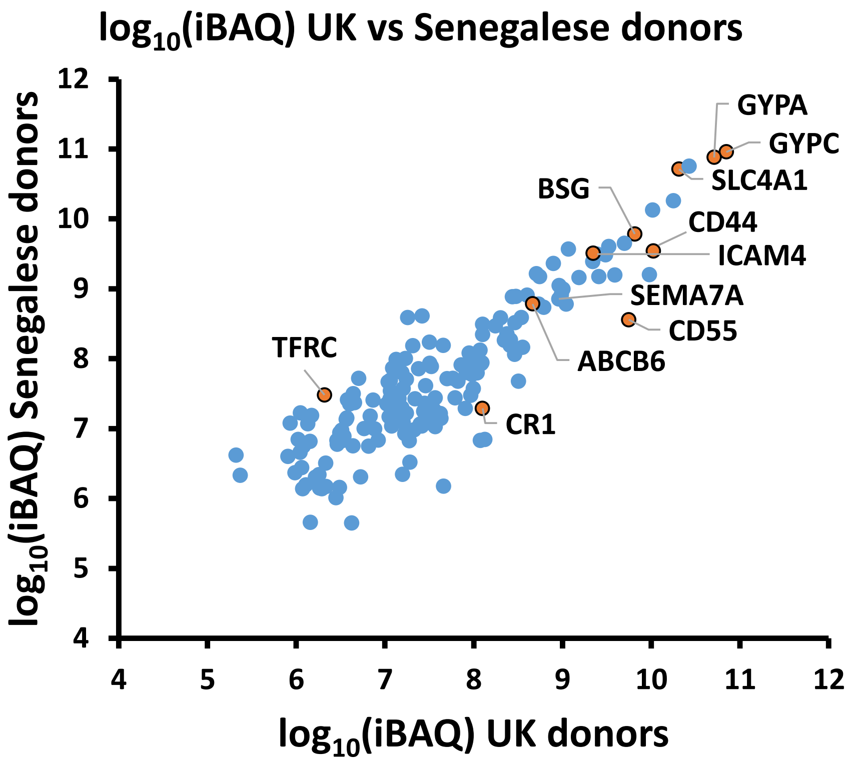

Supplementary Figure 3. Comparison of RBC surface protein abundance between populations. iBAQ abundance values for each protein from UK and Senegalese donors, as shown in Fig. 2a but here with known malaria receptors highlighted.

Supplementary figure 4

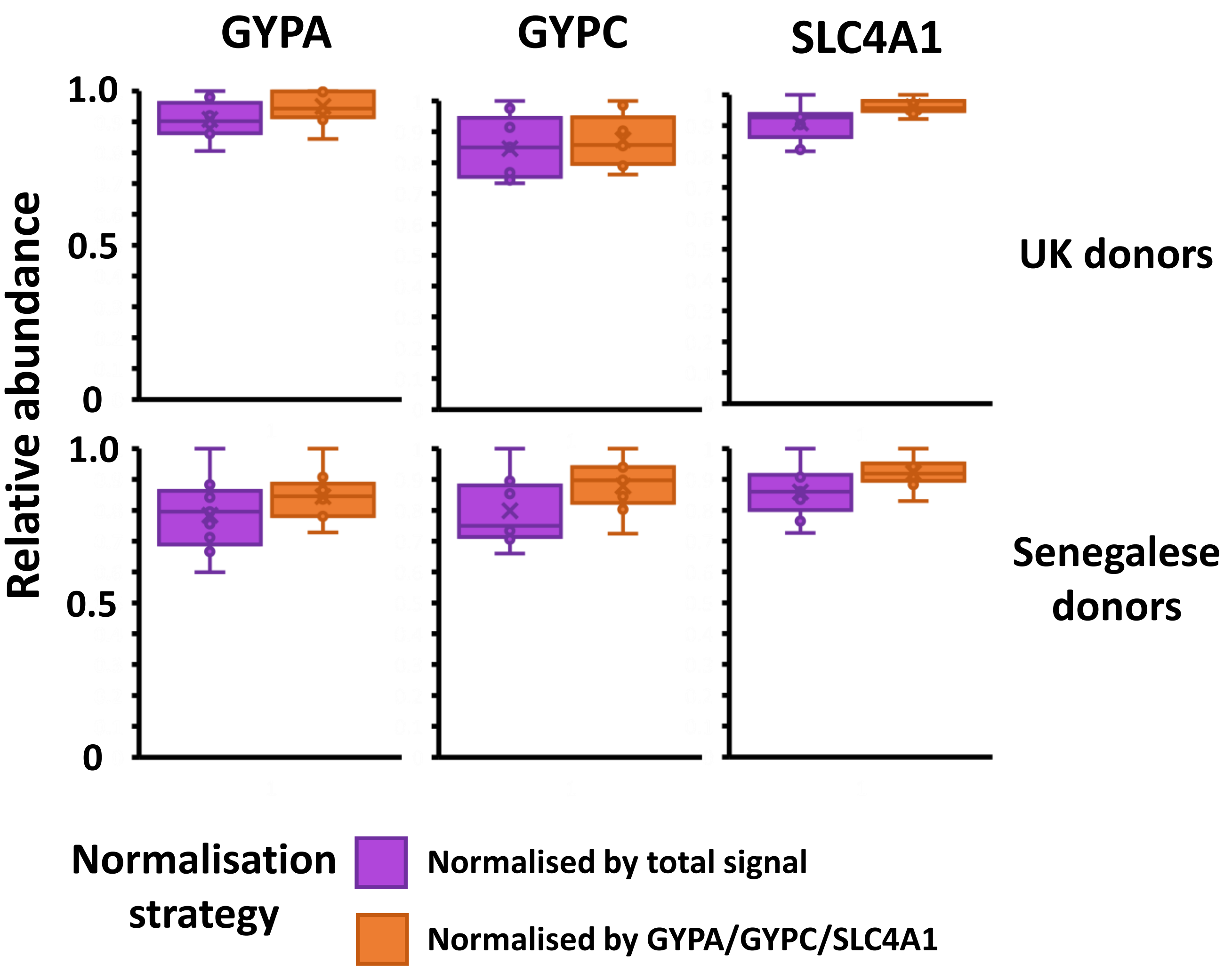

Supplementary Figure 4. Expression of GYPA, GYPC and SLC4A1 molecules are similar across multiple donors, irrespective of normalisation method. Relative abundances of the RBC surface proteins GYPA, GYPC and SLC4A1, using normalisation by two different strategies. Data are plotted as a box and whisker plots showing mean, median and interquartile ranges. n = 9 independent biological samples for each group.

Supplementary figure 5

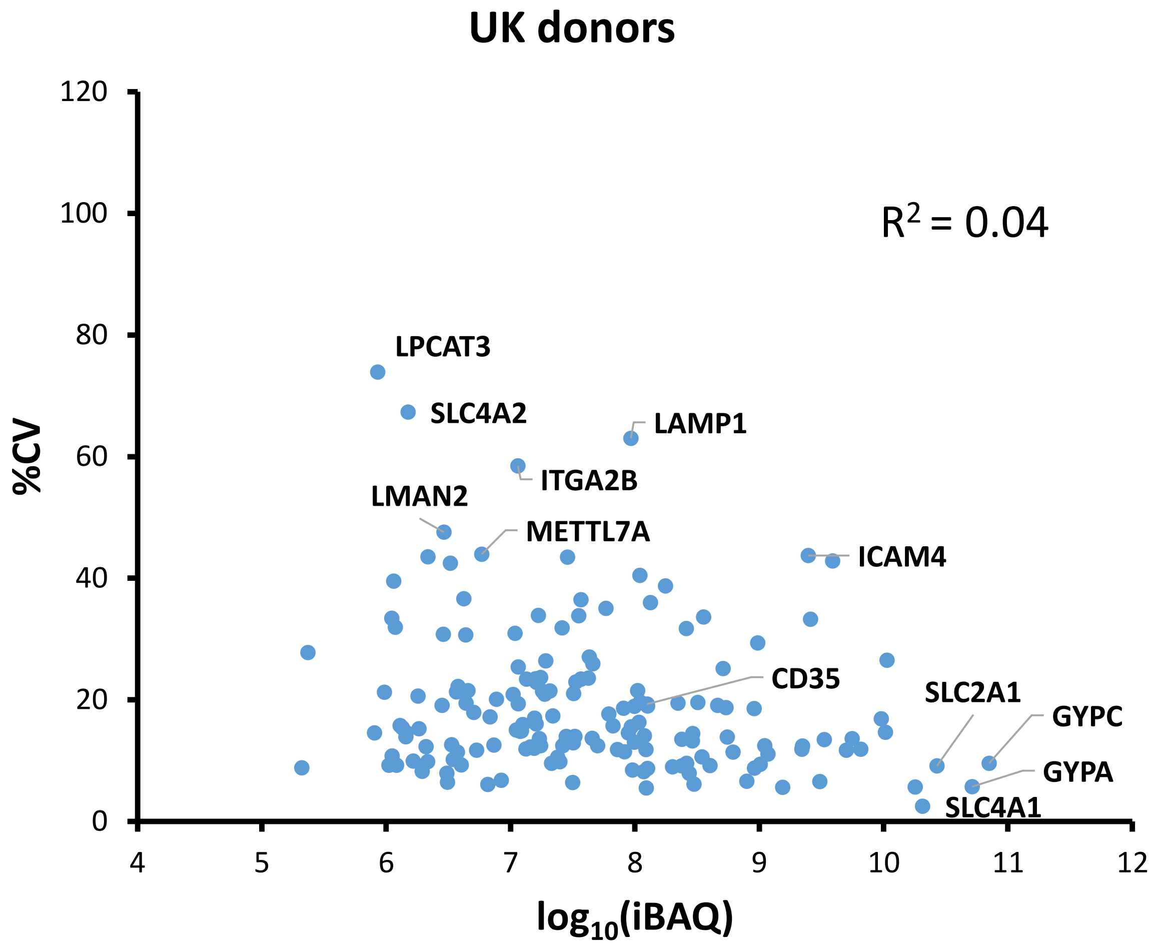

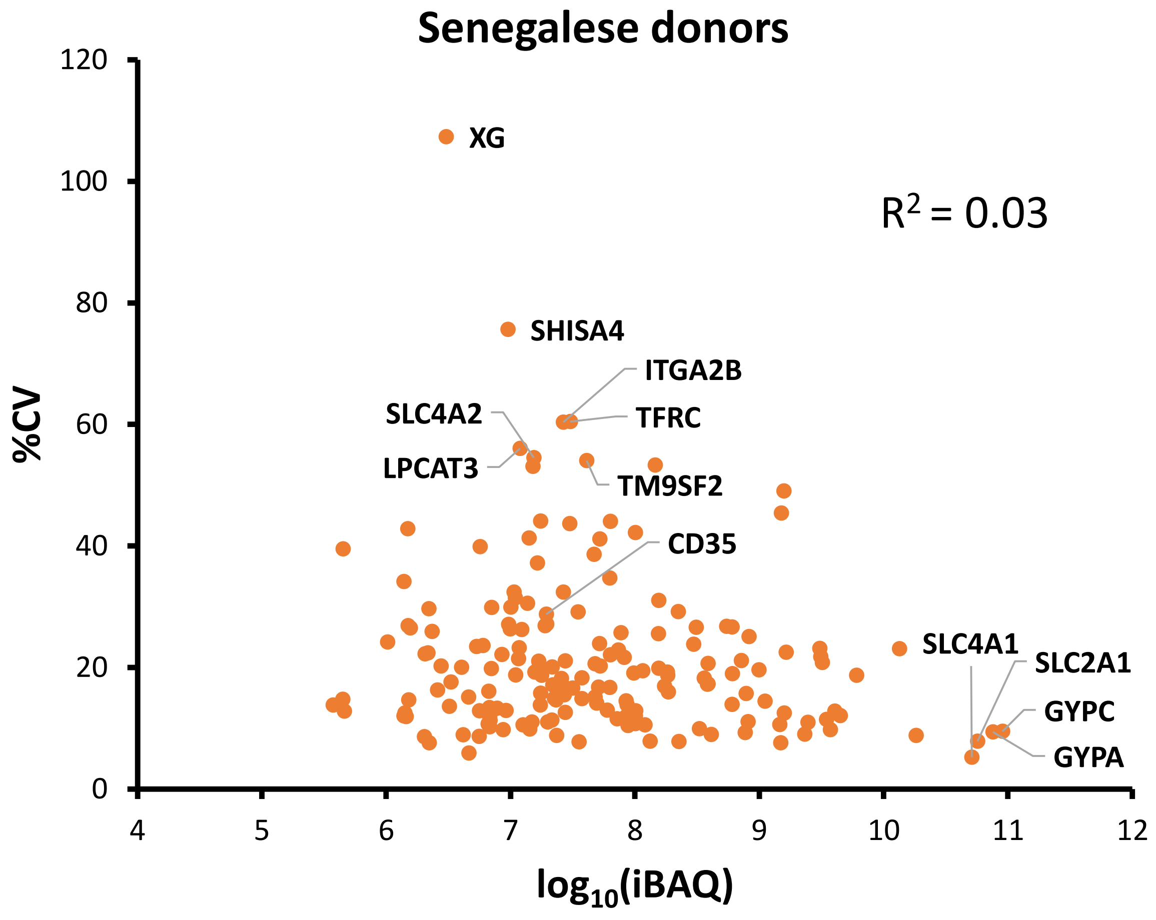

Supplementary Figure 5. Measured variability in protein expression (%CV) does not correlate with protein abundance. Abundance (iBAQ) and %CV values from Fig. 2 and 3 were plotted against one another. Little correlation was seen, apart from low %CV for the four most abundant proteins. For each population these four proteins and the seven most variable proteins are labelled. CD35/CR1 is also labelled for illustration.

Supplementary figure 6

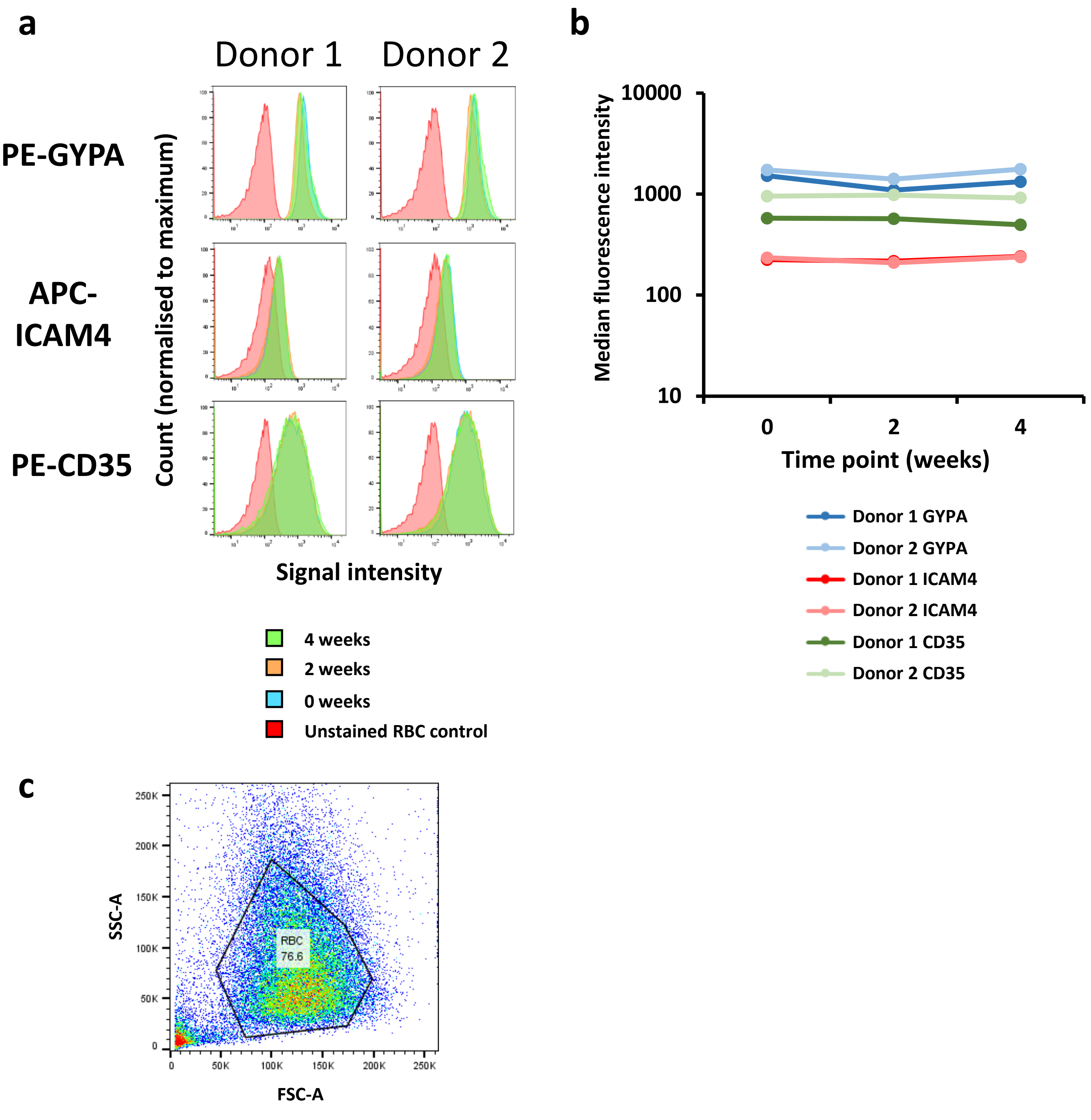

Supplementary Figure 6. Stability of RBC surface protein expression over time assessed by flow cytometry. (a) RBC surface protein expression from two UK donors. Data is displayed as a histogram normalised to the maximum, with fluorescence signal intensity on the x-axis and normalised count on the y-axis. **(b)** The median fluorescence intensity of staining for the three marker proteins illustrated in (a) plotted over time. **(c)** Example of RBC gating strategy based on light scattering properties, shown for deglycerolised RBC.
