# Supplementary material for: Quantitative comparative analysis of human erythrocyte surface proteins between individuals from two genetically distinct populations
Source: Commun Biol. 2019 Sep 20;2:350. doi: 10.1038/s42003-019-0596-y (PMC6754445; doi:10.1038/s42003-019-0596-y)
Supplement: Supplementary file 2 — Description of Supplementary Data [file 42003_2019_596_MOESM2_ESM.docx]

**Description of additional supplementary items – Ravenhill et al 2019.**

**Supplementary Data legends**

**Supplementary data 1.** Serum contaminant analysis by proteomics. Proteins that exhibited increased abundance (based on TMT signal) in un-washed, un-leukodepleted RBC (clusters C1 and C2 in Supplementary Figure. 1D) are shown. Data for each protein is normalised to a maximum signal:noise of 1.

**Supplementary data 2.** Interactive spreadsheet of all data in the manuscript. **(a)** All data for 267 PM proteins identified from either UK or Senegalese donors. Columns include unmodified raw signal:noise values for each protein (columns I-Z), data normalised according to summed signal:noise of GYPA, GYPC and SLC4A1 as described in the Methods and text (columns AC-AT), data further normalised to a maximum of 1 for each protein (columns AW-BN), and derived %CV, iBAQ and significance values (columns BQ-BU). Copy number estimates based on the iBAQ calculations were made using previously published estimates of protein copy number per red blood cell membrane (see Methods for further details). **(b)** 230 PM proteins identified from both UK and Senegalese donors. **(c)** 11 PM proteins only identified in UK donors including Duffy antigen. **(d)** 26 PM proteins only identified in Senegalese donors. **(e)** An interactive plotter displaying the data from Supplementary data 2A. All data in Supplementary data 2A-2E were filtered to include proteins on the ‘sensitive’ RBC cell surface list shown in Supplementary data 3D. **(f)** All quantified and unfiltered proteins, and unmodified raw signal:noise values.

**Supplementary data 3. (a)** 1563 proteins identified either in this study or previous shotgun RBC proteomic experiments, containing at least one indicated Uniprot Subcellular Location term or a positive prediction from TMHMM; **(b)** Details of which studies identified each of the 1563 proteins; **(c)** A ‘stringent’ list of 160 RBC cell surface proteins, requiring identification by our study and at least three previously published studies. **(d)** A ‘sensitive’ list of 240 RBC cell surface proteins, requiring identification by our study and at least one previously published study. **(e)** 27 proteins identified only by the current study with brief annotation.

**Supplementary data 4.** The contribution of blood group proteins with assigned iBAQ values to the total red blood cell surface proteome (from all proteins on the ‘sensitive’ list). Duffy antigen was only quantified in the UK samples, so was not included in this analysis. Unique peptides from GYPB and GYPE were not identified.
