# Supplementary material for: Quantitative comparative analysis of human erythrocyte surface proteins between individuals from two genetically distinct populations
Source: Commun Biol. 2019 Sep 20;2:350. doi: 10.1038/s42003-019-0596-y (PMC6754445; doi:10.1038/s42003-019-0596-y)
Supplement: Supplementary file 3 — Reporting Summary [file 42003_2019_596_MOESM3_ESM.pdf]

nature research

Corresponding author(s): Michael P. Weekes

Last updated by author(s): Jul 8, 2019

Reporting Summary

Nature Research wishes to improve the reproducibility of the work that we publish. This form provides structure for consistency and transparency in reporting. For further information on Nature Research policies, see Authors & Referees and the Editorial Policy Checklist .

Statistics

For all statistical analyses, confirm that the following items are present in the figure legend, table legend, main text, or Methods section.

n/a	Confirmed	
☐	☒	The exact sample size (n) for each experimental group/condition, given as a discrete number and unit of measurement
☐	☒	A statement on whether measurements were taken from distinct samples or whether the same sample was measured repeatedly
☐	☒	The statistical test(s) used AND whether they are one- or two-sided<br>*Only common tests should be described solely by name; describe more complex techniques in the Methods section.*
☒	☐	A description of all covariates tested
☐	☒	A description of any assumptions or corrections, such as tests of normality and adjustment for multiple comparisons
☐	☒	A full description of the statistical parameters including central tendency (e.g. means) or other basic estimates (e.g. regression coefficient) AND variation (e.g. standard deviation) or associated estimates of uncertainty (e.g. confidence intervals)
☐	☒	For null hypothesis testing, the test statistic (e.g. F, t, r) with confidence intervals, effect sizes, degrees of freedom and P value noted<br>*Give P values as exact values whenever suitable.*
☐	☒	For Bayesian analysis, information on the choice of priors and Markov chain Monte Carlo settings
☒	☐	For hierarchical and complex designs, identification of the appropriate level for tests and full reporting of outcomes
☒	☐	Estimates of effect sizes (e.g. Cohen's d, Pearson's r), indicating how they were calculated

Our web collection on statistics for biologists contains articles on many of the points above.

Software and code

Policy information about availability of computer code

Data collection	Thermo Fusion and Thermo Fusion Lumos using Thermo XCalibur v4.1.31.9
Data analysis	Data in this study were analysed with: FlowJo v10 and Excel 2016. Mass spectra were processed using a Sequest-based software pipeline for quantitative proteomics, “MassPike”, through a collaborative arrangement with Professor Steve Gygi’s laboratory at Harvard Medical School. This software is described in detail in the 'Materials and Methods'. For calculation of iBAQ values, MaxQuant 1.6.0.13 was used.

For manuscripts utilizing custom algorithms or software that are central to the research but not yet described in published literature, software must be made available to editors/reviewers. We strongly encourage code deposition in a community repository (e.g. GitHub). See the Nature Research guidelines for submitting code & software for further information.

Data

Policy information about availability of data

All manuscripts must include a data availability statement. This statement should provide the following information, where applicable:

- Accession codes, unique identifiers, or web links for publicly available datasets
- A list of figures that have associated raw data
- A description of any restrictions on data availability

The mass spectrometry proteomics data has been deposited to the ProteomeXchange Consortium (http://www.proteomexchange.org/) via the PRIDE partner repository. Project ascesion number PXD013852, project name ‘Quantitative comparative analysis of human erythrocyte surface proteins between individuals from two genetically distinct populations’. Raw flow cytometry data will be made available on request.

Field-specific reporting

Please select the one below that is the best fit for your research. If you are not sure, read the appropriate sections before making your selection.

- [x] Life sciences
- [ ] Behavioural & social sciences
- [ ] Ecological, evolutionary & environmental sciences

For a reference copy of the document with all sections, see nature.com/documents/nr-reporting-summary-flat.pdf

Life sciences study design

All studies must disclose on these points even when the disclosure is negative.

Sample size
10 primary red blood cell samples taken from UK donors, to match the 10 available tandem mass tags available for mass spectrometry. One sample was excluded due to technical failure, and 9 senegalese samples were taken to match.

Data exclusions
A single UK sample was excluded from further analysis due to a technical failure.

Replication
Flow cytometry was undertaken to confirm the variability of selected markers identified at the red blood cell surface by mass spectrometry, using an independent second set of UK-based samples.

Randomization
Individuals were assigned to sample groups based on their ethnic origin.

Blinding
As the two population samples were collected in distinct geographical locations and processed immediately, blinding was not possible between populations in this study. After collection, samples were linked to the ethnic origin of the donor by a code, without retention of other donor-specific information.

Reporting for specific materials, systems and methods

We require information from authors about some types of materials, experimental systems and methods used in many studies. Here, indicate whether each material, system or method listed is relevant to your study. If you are not sure if a list item applies to your research, read the appropriate section before selecting a response.

Materials & experimental systems

n/a	Involved in the study	
[ ]	[x]	Antibodies
[x]	[ ]	Eukaryotic cell lines
[x]	[ ]	Palaeontology
[x]	[ ]	Animals and other organisms
[ ]	[x]	Human research participants
[x]	[ ]	Clinical data

Methods

n/a	Involved in the study	
[x]	[ ]	ChIP-seq
[ ]	[x]	Flow cytometry
[x]	[ ]	MRI-based neuroimaging

Antibodies

Antibodies used
Antibody Clone|Company|Catalog Number|Lot
FITC-anti-CD45, clone HI-30|Becton-Dickinson|555482|5171102130
APC-anti-CD61, clone VI-PL2|Becton-Dickinson|555754|B216038
APC-anti-ICAM4, Clone # 729632| R&D Biosystems | FAB8397A|AEFM0116081
PE-anti-CD35, clone E11| Miltenyi Biotec|130-099-913| 5171102129
PE-anti-GYPA| Miltenyi Biotec, Clone REA175|130-100-269| 5171010189
PE-anti-CD233(SLC4A1), clone REA368| Miltenyi Biotec|130-105-728|5171117319

Validation
Antibody|Validation
anti-CD45, clone HI-30| Validated against human peripheral blood leukocytes, http://www.bdbiosciences.com/eu/applications/research/stem-cell-research/cancer-research/human/fitc-mouse-anti-human-cd45-hi30/p/555482
anti-CD61, clone VI-PL2|Clone previously validated against human platelets, https://www.biolegend.com/ja-jp/products/purified-anti-human-cd61-antibody-5335
anti-ICAM4, Clone # 729632|Validated against human red blood cells, https://www.rndsystems.com/products/human-icam-4-apc-conjugated-antibody-729632_fab8397a
anti-CD35, clone E11|Validated against human peripheral blood leukocytes, https://www.miltenyibiotec.com/GB-en/products/macs-flow-cytometry/antibodies/primary-antibodies/cd35-antibodies-human-e11-1-11.html#pe:for-30-tests
anti-GYPA, Clone REA175| Validated against CD45 negative human peripheral blood cells| https://www.miltenyibiotec.com/GB-en/products/macs-flow-cytometry/antibodies/primary-antibodies/cd235a-glycophorin-a-antibodies-human-rea175-1-11.html

anti-CD233(SLC4A1), clone REA368| Validated against human peripheral blood erythrocytes, https://www.miltenyibiotec.com/GB-en/products/macs-flow-cytometry/antibodies/primary-antibodies/cd233-antibodies-human-rea368-1-10.html

Human research participants

Policy information about studies involving human research participants

Population characteristics	Healthy participants were selected based on their ethnic background (either Senegalese or from the UK).
Recruitment	Participants volunteered to donate 2-3ml of blood for research.
Ethics oversight	Collection of clinical samples from healthy donors from the Senegalese National Blood Transfusion Center in Dakar, Senegal, and their experimental use were approved by the Ethics Committee of the Ministry of Health in Senegal and by the Institutional Review Board of the Harvard T.H. Chan School of Public Health. For samples derived from donors local to Cambridge, UK, ethical approval was obtained from University of Cambridge Human Biology Research Ethics Committee (HBREC.2016.011). 2-3 ml of whole anticoagulated blood was collected from each donor with informed consent in accordance with the Declaration of Helsinki.

Note that full information on the approval of the study protocol must also be provided in the manuscript.

Flow Cytometry

Plots

Confirm that:

- [x] The axis labels state the marker and fluorochrome used (e.g. CD4-FITC).
- [x] The axis scales are clearly visible. Include numbers along axes only for bottom left plot of group (a 'group' is an analysis of identical markers).
- [x] All plots are contour plots with outliers or pseudocolor plots.
- [x] A numerical value for number of cells or percentage (with statistics) is provided.

Methodology

Sample preparation	RBC were washed, incubated with Fc blocking reagent, stained with specific fluorophore-conjugated antibodies, washed then fixed prior to analysis as described in 'Materials and Methods'. For intracellular staining, a Cytofix/Cytoperm kit (Becton Dickinson) was used.
Instrument	BD LSR Fortessa
Software	BD FACSDIVA<br>FlowJo V10 (FlowJo, LLC)
Cell population abundance	Cells were not sorted - RBC were the major population, identified by light scattering properties.
Gating strategy	Total events >> gate for intact cells by FSC-A vs SSC-A

- [x] Tick this box to confirm that a figure exemplifying the gating strategy is provided in the Supplementary Information.
